# The prevalence of probable sarcopenia and intrinsic capacity impairment among a group of community-dwelling older people in an urban area in Cameroon

**DOI:** 10.1371/journal.pone.0344528

**Published:** 2026-03-20

**Authors:** Marie-Josiane Ntsama Essomba, Régine Mylène Mballa Mba, Jean Jacques Noubiap, Florence Denise Mvondo Lema, Nadine Simo, Maturin Tabue Teguo

**Affiliations:** 1 Department of Internal Medicine and specialties, Faculty of Medicine and Biomedical Sciences, University of Yaounde I, Yaounde, Cameroon; 2 Geriatrics Unit, Yaounde Central Hospital, Yaounde, Cameroon; 3 Division of Cardiology, Department of Medicine, University of California-San Francisco, San Francisco, California, United States of America; 4 Clinical Epidemiology and Aging Team, French West Indies University, French West Indies, France; University Hospital of Padova, ITALY

## Abstract

Although intrinsic capacity serves as an indirect measure of an individual’s functional reserve, whether and in which way it interacts with sarcopenia is still to be addressed in Cameroon. This study aimed to describe the relationship between probable sarcopenia and domains of intrinsic capacity among older people in Cameroon. This cross-sectional study included community-dwelling older aged ≥ 60 years from two senior citizen’s association in Cameroon. Probable sarcopenia was assessed using grip strength and Short Physical Performance Battery (SPPB). Screening for intrinsic capacity(IC) impairment was done using the Integrated Care to Older People(ICOPE) approach. Probable sarcopenia was defined by low handgrip strength and low physical performance. We included variables with a *p*-value < .1 in the multivariable analysis model. The significance level was *P* < .05. We included 108 participants [64.8% female, mean age (standard deviation) 70.4 (6.6) years]. The prevalence of probable sarcopenia was 34.3%. All participants had a positive screening for IC impairment and the main impaired domains were vision (88%), locomotion (61.1%) and cognition (50%). The probable sarcopenia group was likely to be older (72.9 ± 7.9 vs 69.6 ± 6.1 *P* = .018), achieved lower education (*P* = .012) and had frequent history of stroke (*P* = .038). After adjusting for age, sex and comorbidities, participants with impaired vitality (OR3.60, 95%CI1.08–11.94) were likely to have probable sarcopenia. Participants with preserved locomotion (OR 0.12, 95%CI 0.02–0.66) and a lower number of IC domains impaired (OR 0.52, 95%CI 0.29–0.95) were less likely to have probable sarcopenia. With regard to the components of sarcopenia, preserved locomotion (OR 0.01, 95%IC 0.012–0.07) was associated with a higher physical performance. Lower number of IC domains impaired was associated with higher physical performance (OR 0.29, 95%CI0.12–0.76) and higher handgrip strength (OR0.58, 95%CI 0.37–0.92).This study suggest that IC impairment is associated to probable sarcopenia in this group of older adults in Cameroon. Further research on IC trajectories monitoring and incident confirmed sarcopenia as outcome are needed to plan targeted interventions taking into account local resources.

## Introduction

Despite the increase in capacity building, manpower development and implementation of geriatric care in many African countries, the rise in older population and longer lifespans are putting pressure on healthcare systems especially in sub-Saharan Africa (SSA) [1–3]. Several evidence support high prevalence of frailty [4] and its impact on poor quality of life and mortality in many African countries [5–7]. Thus, identifying strategies to prevent or delay transition to frailty and dependency are not only relevant but necessary in a context were policies on aging are still scarce. According to a systematic review published in 2024, the weighted prevalence of sarcopenia in Africa was 25.7% [8]. Although it has been recognized as a disease in the International Classification of Diseases (ICD) for several years [9], the lack of a universal and standardized diagnostic criterion remains one of the main issues for reporting sarcopenia in various settings [10].

The decrease of physical abilities and functional decline that can be caused by sarcopenia, can lead to dependency [11], increased risk of falls [12], poor quality of life [13] as well as increased mortality [14]. Furthermore, sarcopenia leads to a high economic burden and healthcare expenditures [15]. In 2019, the World Health Organisation (WHO) has proposed an Integrated Care to Older People(ICOPE) approach, based on the intrinsic capacity (IC) to tackle rising frailty and dependency in the older population [16]. The domains of IC have been suggested to interact with each other and some of them may overlap with clinical manifestations of sarcopenia. Indeed, IC encompasses domains directly related to muscle function including vitality and locomotion, thus it may capture components that overlap with sarcopenia [16]. Furthermore, handgrip strength is commonly used as an indicator of IC [17].

Although IC serves as an indirect measure of an individual’s functional reserve, whether and in which way it interacts with sarcopenia is still to be addressed in Cameroon. According to a cross-sectional study conducted in Cameroon in 2020, 26% of people aged 55 and over living in an urban setting had a possible sarcopenia [18]. Higher prevalence was found in another group of community-dwellers [7], reaching 52.3% in a hospital-based study conducted in 2021 [19]. Another crucial epidemiological point is that this high prevalence refers to a population having a mean age of 66 years [7,18,19] which is notably younger than the mean age (78 years) in which sarcopenia is typically observed in other settings [20,21]. Given that background, sarcopenia could become a future public health burden in the country. Thus we aim to examine the relationship between probable sarcopenia and domains of IC in a group of older Cameroonians.

## Methods

### Study design and setting

This cross-sectional study was carried out from the 30^th^ July to 1^st^ September 2022 in two senior citizens association of Yaounde, the capital of Cameroon. YOMEHCAM (Our contribution to develop Cameroon Association) and APAN (Aide aux personnes âgées de Nkolbisson Association) are two senior citizens association involved in promoting well being and advocacy for the rights of older people in Cameroon.

### Study population

Participants were recruited during physical activities sessions. Community-dwelling adults aged ≥ 60 years, who provided an informed written consent were included. Participants with severe disability or severe dementia were not included.

### Data collection

#### Demographic and clinical data.

Demographic data included: age (in years), sex, marital status and educational level (illiterate, primary, secondary, university). Clinical data included past medical history including falls history, comorbidities such as hypertension, diabetes, osteoarthritis, obesity and the number of drugs. Polypharmacy was defined as the concomitant use of 5 or more medications.

#### Domains of intrinsic capacity impairment screening.

The IC impairment screening was done as followed:

- Cognition was assessed by the Mini-Cog tool. The Mini-Cog is a quick screening tool and has high sensitivity and specificity to screen for cognitive impairment in older adults across various healthcare settings [22]. A score <3 was considered as impaired cognition.
- Locomotion was assessed by asking the participants to rise from chair five consecutive times without using arms. The incapacity to complete the five-chair rise in less than 14 seconds was considered abnormal [16].
- Vitality was assessed by the Mini-Nutritional Assessment short-form (MNA-SF). The MNA-SF represent a valuable tool for rapid and reliable nutritional screening in the community but also a previously used metric of the vitality domain [17,23]. A score <11 was considered as impaired vitality.
- Psychology was assessed by the 4-item Geriatric Depression Scale (mini-GDS). The mini-GDS 4 yields can be used in screening depression in primary care with a threshold ≥ 1 [24].
- Sensory: Vision was assessed subjectively by asking the participant about difficulties in seeing far, reading, eye diseases or currently under medical treatment for eye disease. If the answer was “yes” to one question, the test was considered abnormal. Audition was assessed by the whispering test for each ear. The examiner stand 1m behind the participant and whisper 2 words at each ear. Failure to repeat one word was considered abnormal.

Any impairment reported for one of the six categories (with vision and hearing considered as two distinct categories) was given a score of 1, otherwise 0. The score was then calculated by adding the number of impaired categories, ranged from 0 to 6. The lower number of IC domains impaired indicates a greater IC.

### Probable sarcopenia assessment

Probable sarcopenia was assessed using the muscle strength and the physical performance. Muscle strength was measured by the handgrip strength on the dominant hand in an individual sitted in upright position, using an electronic Jamar Dynamometer. Participants were instructed to exert maximal force. For each individual, the maximum force (kg) after two measurements was recorded as the participant’s handgrip strength. The cut-off for low handgrip strength was < 20 kg for women and <30 kg for men [25]. The Short Physical Performance Battery (SPPB) was used to assess the physical performance, with a score ranging from 0 to 12 [26]. Low physical performance was considered with a SPPB score ≤ 9. Probable sarcopenia was defined by the presence of low handgrip strength and low physical performance.

### Data analysis

Data were analyzed with the Statistical Package for Social Sciences (SPSS 23.0) for Windows (SPSS, Chicago, Illinois, USA). Quantitative variables were presented as mean and standard deviation (SD) or median and interquantile range (IQR). Categorical variables were presented as frequencies and proportions. Quantitative variables were compared by Student’s T test or U-Mann Witney test when needed. Categorical variables were compared using Chi-squared test or Fisher exact T test as needed. Variables that yielded a *P* < 0.10 by univariable analysis were included in the multivariable logistic regression analysis, which estimated odds ratios (OR) and 95% confidence intervals (CI). In this model, the presence of sarcopenia was the dependent variable and the independent variables were demographic and clinical factors, with the no sarcopenia group as the reference. Additionally, IC was considered the main exposure in separate multivariable logistic regression analysis where each defining component of sarcopenia (muscle strength and physical performance) served as a dependent variable. A *p*-value < 0.05 was satistically significant**..**

### Ethical considerations

This study was approved by the institutional review board of the Yaounde Central Hospital, under the reference number: 17/ACE/CIE/MINSANTE/DHCY/PCE/SG. All participants provided a written consent to participate.

## Results

### Baseline characteristics of participants

108 participants were included, predominantly female (64.8%) with a mean age (standard deviation SD) of 70.4 (6.6) years. As presented in Table 1, the majority of participants was widowed and achieved secondary education. Sixteen (14.8%) of participants have experienced falls. The prevalence of probable sarcopenia was 34.3%. The probable sarcopenia group was likely to be older (72.9 ± 7.9 vs 69.6 ± 6.1 *P* = .018), achieved lower education (*P* = .012) and demonstrated a higher prevalence of past history of stroke (*P* = .038). Other characteristics are presented in Table 1.

**Table 1:** Baseline characteristics of participants.

	Terms	Probable sarcopenia N = 28(%)	No sarcopenia N = 80(%)	All N = 108(%)	*P*
**Sex** ^a^	Male	9 (32.1)	29 (36.3)	38 (35.2)	.695
	Female	19 (67.9)	51 (63.7)	70 (64.8)	–
**Age (years)** ^c^	Mean age(SD)	72.9 (7.2)	69.6 (6.1)	70.4 (6.6)	**<.001**
**Marital status** ^a^	Single	2 (7.1)	11 (13.7)	13 (12)	.339
	Married	10 (35.7)	35 (43.8)	45 (41.7)	–
	Widowed	15 (53.6)	28 (35)	43 (39.8)	–
	Divorced	1 (3.6)	6 (7.5)	7 (6.5)	–
**Education** ^a^	Illiterate	8 (28.6)	8 (10)	16 (14.8)	**.012**
	Primary	13 (46.4)	26 (32.5)	39 (36.1)	–
	Secondary	7 (25)	41 (51.2)	48 (44.4)	–
	University	0 (0)	5 (6.3)	5 (4.6)	–
**Comorbidities(yes)** ^a^	Hypertension	15 (53.6)	39 (48.8)	54 (50)	.661
	Diabetes	3 (10.7)	19 (23.8)	22 (20.4)	.140
	Osteoarthritis	14 (50)	31 (38.8)	45 (41.7)	.299
	Heart failure	2 (7.1)	3 (3.8)	5 (4.6)	.462
	History of stroke	4 (12.3)	2 (2.5)	6 (5.6)	**.038**
**Falls (yes)** ^a^		6 (21.4)	10 (12.5)	16 (14.8)	.252
**Low physcial performance**	Yes	28 (100)	33 (41.2)	61 (56.5)	**<.001**
	No	0 (0)	47 (58.8)	47 (43.5)	

SD standard deviation.

^a^ Categorical variables are shown as number of cases (percentage).

^b^ Continuous variables are shown as median and interquartile range.

^c^ Continuous variables are shown as mean and standard deviation.

All variables with a *p*-value at the 0.1 threshold have been included in the final model.

The *p*-value indicated in bold are statistical significant.

### Relationship between probable sarcopenia and intrinsic capacity

All participants had a positive screening for IC impairment and the median (interquartile range) number of IC domains impaired was 3 [2–3]. The IC characteristics are presented in Table 2. The main domains involved were vision (88%), locomotion (65.7%) and cognition (50%). There were no statistical difference in the probable sarcopenia and the non sarcopenia group in terms of cognition, psychological and sensory impairment. However, the probable sarcopenia group had a higher number of impaired domains (4 vs 3, *P* = 0.038) and was likely to have higher locomotion (92.9% vs 56.2%, *P* < 0.001) and vitality impairment (67.9% vs 36.3%, *P* = 0.004). In the multivariable models (see Table 3), after adjusting for age, sex and comorbidities, participants with preserved locomotion (OR 0.12, 95%CI 0.02–0.66) and a lower number of IC domains impaired (OR 0.52, 95%CI 0.29–0.95) were likely to have a lower prevalence of probable sarcopenia. Impaired vitality (OR 3.60, 95%CI1.08–11.94) was significantly associated with increased prevalence of probable sarcopenia. With regard to the components of sarcopenia, preserved locomotion (OR 0.01, 95%IC 0.012–0.07) and lower number of IC domains impaired (OR 0.29, 95%CI0.12–0.76) were associated with higher physical performance. Participants with lower number of IC domains impaired (OR 0.58, 95%CI 0.37–0.92) had a higher handgrip strength.

**Table 2:** Relationship between probable sarcopenia and impaired IC.

Impaired IC domains	Terms	Probable sarcopenia N = 28(%)	No sarcopenia N = 80(%)	All N = 108(%)	*P*
**Median IC score (IQR)** ^b^		4 (3–4)	3 (2–4)	3 (2–3)	**.038**
**Vision** ^a^	No	1 (3.6)	12 (15)	13 (12)	.177
	Yes	27 (96.4)	68 (85)	95 (88)	–
**Audition** ^a^	No	19 (67.9)	57 (71.3)	76 (70.4)	.735
	Yes	9 (32.1)	23 (28.7)	32 (29.6)	–
**Cognition** ^a^	No	16 (57.1)	38 (47.5)	54 (50)	.380
	Yes	12 (42.9)	42 (52.5)	54 (50)	–
**Locomotion** ^a^	No	2 (7.1)	35 (43.8)	37 (34.3)	**<.001**
	Yes	26 (92.9)	45 (56.2)	71 (65.7)	–
**Vitality** ^a^	No	9 (32.1)	51 (63.7)	60 (55.6)	**.004**
	Yes	19 (67.9)	23 (36.3)	48 (44.4)	–
**Psychology** ^a^	No	18 (64.3)	55 (68.8)	73 (67.6)	.664
	Yes	10 (35.7)	25 (31.3)	35 (32.4)	–

IC intrinsic capacity, IQR interquartile range SD standard deviation.

^a^ Categorical variables are shown as number of cases (percentage).

^b^ Continuous variables are shown as median and interquartile range.

All variables with a *p*-value at the 0.1 threshold have been included in the final model.

The *p*-value indicated in bold are statistical significant.

**Table 3:** Multivariable logistic regression of factors associated with probable sarcopenia and its components.

	Probable sarcopenia			Handgrip strength			Physical performance		
	aOR	95%CI	*P*	aOR	95%CI	*P*	aOR	95%CI	*P*
**Age**	/	/	.152	/	/	.609	/	/	.084
**Sex**	/	/	.564	**2.75**	**1.07-7.03**	**.034**	/	/	.106
**Educational level**	/	/	.299	/	/	.836	/	/	.753
**Stroke**	/	/	.484	/	/	.993	/	/	.999
**Vitality**	**3.60**	**1.08-11.94**	**.036**	/	/	.271	/	/	.057
**Locomotion**	**0.12**	**0.02-0.66**	**.014**	/	/	.352	**0.01**	**0.012-0.07**	**<.001**
**Number of impaired IC domains**	**0.52**	**0.29-0.95**	**.029**	**0.58**	**0.37-0.92**	**.020**	**0.29**	**0.12-0.76**	**.012**

aOR adjusted odds ratio, CI confidence intervall, IC intrinsic capacity.

## Discussion

In this cross-sectional study in a group of community-dwelling older adults, we examined the association between IC, sarcopenia as well as its components. Probable sarcopenia was highly prevalent, affecting approximately one-third of participants. All individuals were screened positive for impairment of at least one domain of IC, with visual, cognitive and locomotor domains being the most frequently affected. Older age, low education and history of previous stroke were more common among participants with probable sarcopenia. After adjustement for age, sex and comorbidities, impairment in the vitality domain was independently associated with a higher likelihood of probable sarcopenia, whereas preserved locomotion and lower number of IC domains impaired were associated with lower odds. With regard to the components of sarcopenia, preserved locomotion and lower number of IC domains impaired were associated with greater physical performance and higher handgrip strength.

Consistent with previous research, sarcopenia was strongly associated with higher number of IC domains impaired and with impairment in specific IC domains, particularly locomotion and vitality. In hospitalized older patients [27] and in community-dwelling octogenarians [28] lower IC composite scores and impairments in locomotion, vitality and cognition are associated with higher odds of sarcopenia. Similarly, studies conducted in disease-specific populations, such as older adults with type 2 diabetes has demonstrated that a greater number of impaired IC domains is associated with increased risk of sarcopenia, with locomotion and sensory domains playing a major role [29]. These results are further supported by a systematic review reporting robust interrelation between decline in IC domains, particularly in locomotion and vitality and sarcopenia [30]. Beyond its geographical context, this study contributes by simultaneously evaluating IC domains alongside distinct components of sarcopenia within a single community-based population, thereby extending existing findings beyond selected clinical or disease-specific settings. While our results corroborate this existing body of evidence, they extend prior work in several important ways. First, unlike studies focusing on highly selected populations (e.g., hospitalized patients, octogenarians or individuals with specific chronic diseases) our analysis was conducted in a community-dwelling population, potentially capturing earlier stages of functional decline. Secondly, by examining not only probable sarcopenia as a binary condition but also its components we provide an understanding of how different aspects of muscle function may relate to IC.

With regard to the prevalence of probable sarcopenia, our findings differ from those of previous studies conducted in Cameroon. In 2021, 26% of adults aged ≥ 55 years in an urban setting had possible sarcopenia [18]. In another study involving 403 older community-dwellers, the prevalence of sarcopenia was 47.9% [7]. Our prevalence was also far from the 53% reported in a group of hospitalized patients aged 55 years and over [19]. Our prevalence is higher in comparison to those observed in other African countries. In 2024, Ajuaonuma et al reported a prevalence of 21.1% in a group of retirees in Nigeria [31]. In a group of Gambian aged between 40 and 75 years, the prevalence of sarcopenia varied depending on the definition used; in men 20% and 19% and in women 45% and 10% [32]. Although it has been recognized as a disease in the International Classification of Diseases (ICD) for several years [9], the lack of a universal and standardized diagnostic criteria for sarcopenia remains one of the main issues in assessing this condition. As expected, the different criteria used have led to a very heterogeneous prevalence of sarcopenia, even in the same settings. These discrepancies are due to several approaches for the diagnosis of sarcopenia, from those integrating muscle strength, physical performance, and body composition parameters to those using only body composition. Furthermore, several societies and organizations has proposed definitions which are region specific in Western [33] and Asian [34] countries but to date, no operational definition of sarcopenia exists. Another concern when dealing with sarcopenia in SSA, is the lack of specific cut-off values in the muscle mass and muscle strength assessment for the Black Africans sub-populations.

We found that impaired vitality was independently associated with an increased prevalence of probable sarcopenia. This association between vitality and sarcopenia are in line with previous research. In a study conducted in a group of 599 octogenerians in China, those with possible sarcopenia or sarcopenia were more likely to have decline in vitality domain [28]. In another cross-sectional study investigating the relationship between sarcopenia and IC in hospitalized older adults, the sarcopenia group had lower scores in locomotion and vitality domains than the non-sarcopenia group and a strong association was found between impaired vitality and sarcopenia [27]. In a cross-sectional study of a large community-dwelling population, almost all definitions of sarcopenia were associated with a poor nutritional status measured by the MNA [35]. According to a recent systematic review, vitality, locomotion, and cognition are key functional areas of IC linked to sarcopenia [30]. Impaired vitality reflects nutritional and energy-related disorders. Our findings support the notion that impaired vitality may represent a key pathway linking intrinsic capacity impairment to declines in muscle strength and physical performance in older adults. From a pathophysiological point of view, both malnutrition and sarcopenia share many components. With regard to nutritional status, vitality domain is considered as the underlying physiological determinant of IC, resulting from the interaction between multiple physiological systems, reflected in metabolism, neuromuscular function and immune and stress response functions of the body [36]. Beside that, sarcopenia arises from the complex interaction between several factors including chronic inflammation, mitochondrial dysfunction, impaired neuromuscular signaling and reduced protein synthesis [37–40]. Poor nutritional status is common among older people in Cameroon [41] and the rising prevalence of poor physical performance is also a matter of concern [42,43].

We found that preserved locomotion was independently associated with a lower prevalence of probable sarcopenia and higher physical performance. However in multivariable models including probable sarcopenia, locomotion and IC domains, effect estimates showed instability in direction, likely due to collinearity between closely related measures of physical function. These findings should therefore be interpreted with caution and do not suggest true inverse associations. Few studies reported the relationship between IC and physical performance. Individuals with higher IC were less likely to report recent falls, with locomotion being an independently associated domain in a group of octogenerians [44]. Tay et al found that IC was significantly associated with fitness performance, independent of age and gender [45]. Other studies have focused on the relationship between IC and physical activity which although conceptually differs from physical performance, remains closely related. In a study involving a group of healthy older adults, higher moderate-to-vigorous physical activity levels were associated with higher IC mobility, vitality and psychological domain in active versus inactive individuals [46]. Furthermore, the authors found that the inactive category experienced a significant or nearly significant decline in IC, mobility and psychology, while no significant change was observed in the active group [46].One of the most prominent change associated to sarcopenia is decline in locomotion [11]. This decline is further exacerbated by reduced physical activity which may accelerate muscle loss. Older adults may therefore exhibit slow gait and poor balance, thereby impacting physical performance and increasing the risk of recurrent falls [44]. Although physical activity was not directly measured in our study, the context of recruitment suggests a predominantly active population. Indeed, participants in our study were involved in physical activity sessions organized by their associations. This behavioural context may have contributed to the presentation of locomotion and physical performance observed in our study. However, a substantial proportion of participants met the criteria for probable sarcopenia. Several hypothesis may explain this apparent paradox. First, physical activity sessions may be insufficient in intensity or resistance loading to counteract impaired muscle function. Activities in these associations usually focus on maintaining mobility and social engagement and may not adequatly stimulate muscle strength. In addition, the high prevalence of cerebrovascular disease among participants with probable sarcopenia may compromise muscle strength and physical performance.

In our study, lower number of IC domains impairedwas independently associated with a lower prevalence of sarcopenia. It was also independently associated with higher handgrip strength and higher physical performance. Our results are in line with previous studies. Zhu et al found that higher IC composite score was an independent risk factor for sarcopenia and deficits in its associated components namely handgrip strength, skeletal muscle mass and gait speed [27]. Handgrip strength asymmetry and weakness were both significantly associated with an increased risk of IC impairment in older Chinese adults, with the simultaneous presence of both conditions conferring the highest risk. Additionally, a substantial association was observed between handgrip strength status and each domain of IC [47]. In a cross-sectional study conducted in Colombia, participants with optimal handgrip strength had better IC than weak older adults, including both men (OR 0.62, 95%CI 0.53–0.71) and women (OR 0.79, 95% CI 0.68–0.92) [48]. The mechanism for the association between IC and sarcopenia is not yet completly understood. However, sarcopenia is characterized by age-related loss of muscle mass and function which overlap with some domains of IC.

While the IC domains are separate entities and interventions may be domain specific, our findings are in line with previous studies suggesting significant interactions between domains notably vitality and locomotion. Studies on relevant interventions are lacking. Nevertheless, this is an active area of research with promising results. With regard to sarcopenia, current evidence suggests that protein supplementation is effective in improving muscle strength and muscle mass when used as an adjunct to resistance exercise training [49]. Multidomains lifestyle interventions on nutrition, cognition and mobility may be effective in reversing sarcopenia and improving muscle mass and function among community-dwelling older [50]. In another randomized control trial, 6-month active combination of psychological, nutrition and exercise interventions improved sarcopenia indices [51].

### Strength and limitations

To the best of our knowledge, this is the first study to examine the relationship between probable sarcopenia and IC in Cameroon, highlighting the impact of vitality, locomotion and number of impaired IC domains in sarcopenia, handgrip strength and physical performance. Our findings suggest that despite their involvement in senior associations our participants did not demonstrate a lower prevalence of locomotion impairment relative to other older populations in Cameroon. This suggests that participation in activity groups alone may not be sufficient to preserve locomotion or that underlying factors such as comorbidities, environmental constraints or early functional decline may still contribute significantly. However, we acknowledge several limitations. First, the cross-sectional design did not allow us to determine the causality between IC impairment and sarcopenia. Second, our sample size is not large enough to generalize our findings. Third, possible selection bias as we choose to recruit among a group of active individuals, thus older people living with frailty are probably underrepresented in this population. Finally, comorbidities were not collected using a standardized index limiting analysis to participants conditions and muscle mass was not measured to confirm the diagnosis of sarcopenia.

## Conclusion

This study showed a high prevalence of probable sarcopenia and IC impairment in a group of active older Cameroonians. Our findings enable a nuanced evaluation of the relationship between muscle function components and IC. Further research on IC trajectories monitoring and incident confirmed sarcopenia as outcome are needed to plan targeted interventions taking into account local resources.

## Supporting information

S1 File Inclusivity-in-global-research-questionnaire. (DOCX)
